# Comparative Evaluation of Broth Microdilution With Disc Diffusion and VITEK 2 for Susceptibility Testing of Colistin on Multidrug-Resistant Gram-Negative Bacteria

**DOI:** 10.7759/cureus.50894

**Published:** 2023-12-21

**Authors:** Anita, Ritu Kumari, Kumar Saurabh, Santosh Kumar, Namrata Kumari

**Affiliations:** 1 Microbiology, Indira Gandhi Institute of Medical Sciences, Patna, IND; 2 Emergency Medicine, Indira Gandhi Institute of Medical Sciences, Patna, IND

**Keywords:** gram-negative bacteria, multidrug resistant, colistin, vitek 2, disc diffusion

## Abstract

Background

The rise of antibiotic resistance, particularly in Gram-negative bacteria, poses a significant global health threat. Colistin, a last-resort antibiotic, has witnessed renewed use. However, accurate susceptibility testing for colistin is challenging, with various methods available, leading to potential discrepancies. Ensuring reliable testing is crucial for effective patient treatment and antimicrobial stewardship. This study addresses the need to compare different colistin susceptibility testing methods, providing insights into their accuracy and relevance in clinical settings.

Methods

In this one-year prospective observational cross-sectional study conducted at Indira Gandhi Institute of Medical Sciences (IGIMS), Bihar, India, a tertiary care hospital from July 2021 to June 2022, we aimed to evaluate the concordance between two widely used methods, VITEK 2 and Disc Diffusion, for antibiotic susceptibility testing in clinical multidrug-resistant Gram-negative bacterial isolates. These isolates, including species like *Klebsiella pneumoniae*, *Acinetobacter baumannii*, *Klebsiella oxytoca*, *Pseudomonas aeruginosa*, *Citrobacter freundii*, and *Escherichia coli*, were isolated from various clinical specimens. After rigorous species-level identification and quality control measures, antibiotic susceptibility testing was performed using both methods, and their agreement was assessed through Percentage Agreement analysis.

Results

In our study, we isolated and identified bacterial isolates from 105 patients, with a mean age of 47.30 years, demonstrating a wide age range. Pus samples were the most common type (25.7%), and *K. pneumoniae* was the most prevalent organism (45.7%). Antibiotic resistance patterns revealed significant challenges in treating infections caused by *K. pneumoniae* and *A. baumannii*, with resistance rates exceeding 70% for certain antibiotics. Among the 48 isolates of *K. pneumoniae*, the agreement was 93.8%, with 89.6% being sensitive and 6.3% being resistant by Disc Diffusion, while VITEK 2 indicated 0% resistance. *E. coli* isolates (n=21) had an agreement of 90.5%, with 90.5% sensitivity and 9.5% resistance by Disc Diffusion, and no resistance by VITEK 2.

Conclusion

The comparative analysis of antibiotic susceptibility testing methods reveals the superior performance of the VITEK 2 system, particularly in sensitivity and negative predictive value, emphasizing its potential as a reliable tool for guiding antibiotic therapy decisions.

## Introduction

Multidrug-resistant Gram-negative bacteria represent a formidable challenge in contemporary healthcare settings. These microorganisms, including but not limited to *Escherichia coli*, *Klebsiella pneumoniae*, *Acinetobacter baumannii*, and *Pseudomonas aeruginosa*, have developed resistance mechanisms that render them impervious to a wide array of antibiotics. This rise in multidrug resistance threatens to compromise the efficacy of traditional treatment regimens and leaves healthcare providers with limited therapeutic options [1,2].

In this context, colistin, a polymyxin antibiotic, has re-emerged as a crucial antibiotic of last resort. Colistin exhibits bactericidal activity by disrupting the integrity of the outer membrane of Gram-negative bacteria, a mechanism distinct from most other antibiotics. While colistin was introduced decades ago, its use had been largely curtailed due to concerns about nephrotoxicity and neurotoxicity. However, as the incidence of multidrug-resistant Gram-negative infections continues to escalate, colistin has been revived as a vital treatment option, despite these adverse effects [3-5].

Colistin resistance, primarily mediated by chromosomal mutations and plasmid-encoded resistance determinants such as MCR genes, has now become a global concern. The rapid emergence of colistin-resistant strains poses a dire threat to healthcare, emphasizing the urgency of accurate susceptibility testing methods. Accurate testing is essential to guide clinical decisions, prevent the inappropriate use of colistin, and conserve its efficacy as a last-resort antibiotic [5,6].

Traditional methods of susceptibility testing, such as Disc Diffusion, have been a mainstay in clinical laboratories for decades. However, these methods have inherent limitations, particularly when assessing colistin susceptibility, due to variations in diffusion patterns and interpretive challenges. Broth Microdilution, an established reference method, offers higher precision but is labor-intensive and time-consuming, making it less suitable for rapid clinical decision-making. European Committee on Antimicrobial Susceptibility Testing (EUCAST) provides standardized guidelines for antimicrobial susceptibility testing in Europe. For colistin, EUCAST recommends the determination of the minimum inhibitory concentration (MIC) using Broth Microdilution methods. MIC values are interpreted based on breakpoints defined by EUCAST. EUCAST has specific breakpoints for colistin against various bacterial species. The breakpoints categorize isolates as susceptible, intermediate, or resistant. The interpretation takes into account the clinical efficacy of colistin against the specific pathogen. Clinical and Laboratory Standards Institute (CLSI) is a global organization that provides clinical laboratory standards for antimicrobial susceptibility testing. Similar to EUCAST, CLSI recommends the determination of MIC for colistin using Broth Microdilution methods. CLSI has breakpoints for colistin that guide the interpretation of susceptibility. These breakpoints are organism-specific and take into consideration the pharmacokinetics/pharmacodynamics of colistin against different bacteria [7,8].

The introduction of automated systems like VITEK 2 has promised to address some of these challenges. These systems offer the advantages of speed and efficiency while maintaining accuracy, making them appealing for routine susceptibility testing. However, their performance in accurately assessing colistin susceptibility, especially in the presence of multidrug-resistant mechanisms, requires rigorous evaluation [9-12].

This study aimed at a comparative evaluation of Broth Microdilution with Disc Diffusion and VITEK 2 for susceptibility testing of colistin on multidrug-resistant Gram-negative bacteria.

## Materials and methods

Study design

This prospective observational cross-sectional study was designed to evaluate the concordance between two widely used methods, VITEK 2 and Disc Diffusion, for assessing antibiotic susceptibility in clinical bacterial isolates (multidrug-resistant Gram-negative bacteria). The research was conducted under the Department of Microbiology, IGIMS, Bihar a tertiary care hospital (with a bed capacity of 1200), for one year between July 2021 and June 2022 (IEC approval number: 254/IEC/IGIMS/2021). The facility caters to a diverse patient population. A monthly average of 2000 samples are processed for microbial testing, with an emphasis on identifying and culturing. Among these, around 300 samples are specifically dedicated to bacterial culture. These samples encompassed various types, including pus, urine, blood, endotracheal tube (ETT), and others, reflecting the wide spectrum of infections encountered in clinical practice.

Inclusion and exclusion criteria

Patients aged 18 years and older who presented in various inpatient or outpatient departments with clinically suspected infections, and having multidrug-resistant Gram-negative bacterial isolates (including *K. pneumoniae*, *A. baumannii*, *Klebsiella oxytoca*, *P. aeruginosa*, *Citrobacter freundii*, and *E. coli*) from clinical specimens (such as ascitic fluid, bile, blood, central line, corneal, drainage, ETT, high vaginal swab, liver abscess (pus), pleural fluid, pus, sputum, tissue, tissue biopsy, tracheal, urine, and vitreous humor) were included in this study. Participants were required to have provided informed consent for sample collection. The exclusion criteria for this study were all Gram-positive bacterial species, including cocci like *Staphylococcus aureus* and *Streptococcus pneumoniae*. Additionally, samples with insufficient quantity or compromised integrity, and incomplete or missing medical records essential for the study were excluded from the analysis.

Bacterial isolate collection

Upon collection, each clinical specimen was processed using standard laboratory protocols (such as labeling and documentation; transport and storage biochemical; specimen preparation; microbiological culture; microscopic examination; biochemical tests; antibiotic susceptibility testing; and data recording) [7]. The isolation of Gram-negative bacterial colonies from the clinical sample involved streaking the specimen onto appropriate agar plates to ensure the purity and vitality of the isolates. The isolated colonies underwent species-level identification through microbiological and biochemical tests, including Gram staining, biochemical profiling, and, VITEK MS (BIOMERIEUX). This step aimed to confirm the identity of the isolates and classify them into specific Gram-negative bacterial species (e.g., *K. pneumoniae*, *A. baumannii*, etc.). Following identification, the Gram-negative bacterial isolates were preserved to maintain their viability for subsequent testing.

Preservation methods for the collected Gram-negative bacterial isolates were meticulously executed to maintain their viability and integrity. Cryovials, specifically designed for long-term storage of bacterial cultures, were utilized to ensure a secure and standardized containment system. The isolates were labeled with precision, detailing pertinent information for accurate record-keeping. Storage temperature was consistently maintained at an optimal level, typically at -80°C, to inhibit bacterial degradation and preserve their genetic and phenotypic characteristics effectively. For subsequent testing, a controlled and standardized sub-culturing process was implemented using American Type Culture Collection (ATCC) strains such as *E. coli* (ATCC 25922) and *P. aeruginosa* (ATCC 27853). This involved periodic sub-culturing (typically on a monthly basis or as needed based on the growth characteristics and storage duration of the bacterial isolates) to maintain the vitality of the isolates and ensure that they remained viable for downstream analyses.

Antibiotic susceptibility testing

Two distinct methods were employed for antibiotic susceptibility testing (including ampicillin, cefepime, cefotaxime, ciprofloxacin, amikacin, cotrimoxazole, gentamicin, imipenem, meropenem, ceftazidime, piperacillin-tazobactam, minocycline, aztreonam, tobramycin, polymyxin-B, amoxyclav, and nitrofurantoin) of the bacterial isolates. The VITEK 2 automated system (bioMérieux) was utilized for determining the antibiotic susceptibility profiles of the isolates. This method relies on automated analysis and categorization of bacterial responses to specific antibiotics. The results were categorized as “Sensitive” or “Resistant,” and the percentages of sensitivity and resistance were calculated for each bacterial species. In parallel, the Disc Diffusion method was employed as an alternative approach for antibiotic susceptibility testing. This method involves the placement of antibiotic discs on Muller Hilton Agar inoculated (for 16 to 18 hours) with bacterial isolates. The zone diameters around each disc were measured and interpreted based on established clinical breakpoints. Results were categorized as “Sensitive” or “Resistant,” and the percentages of sensitivity and resistance were calculated for each bacterial species.

Statistical analysis

Descriptive statistics were used to summarize the demographic and clinical characteristics of the patient population, including mean age, gender distribution, and ward distribution. Frequency distributions were generated to display the distribution of bacterial isolates by species (e.g., *K. pneumoniae*, *A. baumannii*, etc.) and their antibiotic susceptibility profiles (sensitive, intermediate, resistant). To assess the agreement and discrepancies between two different methods of antibiotic susceptibility testing (Kirby-Bauer Disc Diffusion method and the VITEK 2 system), comparative analysis was performed using Percentage Agreement. This was calculated as the percentage of isolates for which both methods yielded concordant results (either both “Sensitive” or both “Resistant”).

Ethical considerations

The ethical approval was obtained (IEC approval number: 254/IEC/IGIMS/2021). To safeguard patient privacy and uphold ethical standards, all patient-related data associated with the isolates were anonymized. This entailed the removal of any personally identifiable information and the assignment of unique codes or identifiers to each isolate. Anonymization was imperative to protect patient confidentiality and adhere to ethical guidelines.

## Results

In our study, a total of bacterial isolates from 105 patients were collected and identified. The mean age of the participants was 47.30 years with a standard deviation of ± 21.46 years, indicating a wide age range in the sample. Regarding the distribution of sexes, the study included 69 males (65.7%) and 36 females (34.3%), demonstrating a slight male predominance in the sample population. In terms of ward allocation, the majority of patients were from the outpatient department (OPD), accounting for 44 cases (41.9%). Other wards with notable representation included the intensive care unit (ICU) with 12 cases (11.4%) and the postoperative ward (PVT) with three cases (2.9%). The rest of the wards had relatively smaller proportions. Sample type analysis revealed that pus samples were the most common, constituting 27 cases (25.7%), followed by urine samples with 21 cases (20%). Other sample types, such as ETT and sputum, also had substantial representation. The study identified several organisms responsible for infections, with *K. pneumoniae* being the most prevalent at 45.7%. *A. baumannii* accounted for 17.1% of cases, while other organisms like *E. coli* and *P. aeruginosa* were responsible for smaller proportions of infections (Table 1).

**Table 1:** Baseline characteristics bacterial isolates of the patients (N=105). OPD: outpatient department

Variables	Frequency	%
Mean age (in years)	47.30 ± 21.46	
Sex		
Male	69	65.7
Female	36	34.3
Ward/OPD type		
Critical care medicine	45	42.9
Dental	12	11.4
Extended high-dependency unit	9	8.6
Emergency intensive care unit	7	6.7
General medicine	7	6.7
General surgery	6	5.7
High dependency unit	4	3.8
Intensive care unit	3	2.9
Medical intensive care unit	3	2.9
Neurology	3	2.9
Outpatient department	2	1.9
Pediatric intensive care unit	1	1.0
Private ward	1	1.0
Respiratory	1	1.0
Urology	1	1.0
Sample Type		
Ascitic fluid	27	25.7
Bile	21	20.0
Blood	13	12.4
Central line	12	11.4
Corneal	9	8.6
Drainage	6	5.7
Endotracheal tube	4	3.8
High vaginal swab	2	1.9
Liver abscess (pus)	2	1.9
Pleural fluid	2	1.9
Pus	1	1.0
Sputum	1	1.0
Tissue	1	1.0
Tissue biopsy	1	1.0
Tracheal	1	1.0
Urine	1	1.0
Vitreous humor	1	1.0
Gram-Negative Bacteria		
Klebsiella pneumoniae	48	45.7
Acinetobacter baumannii	21	20.0
Klebsiella oxytoca	18	17.1
Pseudomonas aeruginosa	11	10.5
Citrobacter freundii	6	5.7
Escherichia coli	1	1.0

The analysis of antibiotic resistance patterns among various organisms revealed significant challenges in treating infections caused by *K. pneumoniae* and *A. baumannii*, which displayed high resistance rates exceeding 70% to antibiotics such as ampicillin, cefepime, and cefotaxime. In contrast, *K. oxytoca* showed notable sensitivity to most antibiotics tested, except for ciprofloxacin, where resistance was observed in 17.8% of cases. *P. aeruginosa* exhibited resistance to multiple antibiotics. Both *C. freundii* and *E. coli* exhibited varying degrees of resistance. Amikacin and gentamicin showed relatively higher sensitivity rates, suggesting potential effectiveness in treating infections. Carbapenems, imipenem, and meropenem displayed some effectiveness but with significant resistance in *A. baumannii*. Cotrimoxazole showed varying resistance rates among organisms, indicating limited utility. Polymyxin-B and nitrofurantoin demonstrated sensitivity in most cases, while aztreonam and tobramycin exhibited resistance (Table 2).

**Table 2:** Distribution of antibiotic susceptibility for Gram-negative bacteria in isolates of patients.

Antibiotics	Total	Gram-negative bacteria [Frequency (%)]					
		*Klebsiella pneumoniae*	*Acinetobacter baumannii*	*Klebsiella oxytoca*	*Pseudomonas aeruginosa*	*Citrobacter freundii*	*Escherichia coli*
Ampicillin							
Resistant	76 (72.4)	48 (63.2)	0 (0.0)	6 (7.9)	0 (0.0)	1 (1.3)	21 (27.6)
Not done	29 (27.6)	0 (0.0)	18 (62.1)	0 (0.0)	11 (37.9)	0 (0.0)	0 (0.0)
Cefepime							
Resistant	76 (72.4)	48 (63.2)	0 (0.0)	6 (7.9)	0 (0.0)	1 (1.3)	21 (27.6)
Not done	29 (27.6)	0 (0.0)	18 (62.1)	0 (0.0)	11 (37.9)	0 (0.0)	0 (0.0)
Cefotaxime							
Resistant	94 (89.5)	48 (51.1)	18 (19.4)	6 (6.5)	0 (0.0)	1 (1.1)	21 (22.6)
Not done	11 (10.5)	0 (0.0)	0 (0.0)	0 (0.0)	11 (100.0)	0 (0.0)	0 (0.0)
Ciprofloxacin							
Sensitive	4 (3.8)	3 (75.0)	0 (0.0)	0 (0.0)	0 (0.0)	0 (0.0)	1 (25.0)
Resistant	101 (96.2)	45 (44.6)	18 (17.8)	6 (5.9)	11 (10.9)	1 (1.0)	20 (19.8)
Amikacin							
Sensitive	27 (24.5)	12 (44.4)	1 (3.7)	3 (11.1)	2 (7.4)	1 (3.7)	8 (29.6)
Resistant	78 (75.5)	36 (46.2)	17 (21.8)	3 (3.8)	9 (11.5)	0 (0.0)	13 (16.7)
Cotrimoxazole							
Sensitive	22 (20.4)	12 (54.5)	4 (18.2)	1 (4.5)	0 (0.0)	0 (0.0)	5 (22.7)
Resistant	72 (66.7)	36 (50.0)	14 (19.4)	5 (6.9)	0 (0.0)	1 (1.4)	16 (22.2)
Not done	11 (10.2)	0 (0.0)	0 (0.0)	0 (0.0)	11 (100.0)	0 (0.0)	0 (0.0)
Gentamicin							
Sensitive	19 (17.3)	7 (36.8)	2 (10.5)	1 (5.3)	1 (5.3)	0 (0.0)	8 (42.1)
Resistant	86 (78.9)	41 (47.7)	16 (18.6)	5 (5.8)	10 (11.6)	1 (1.2)	13 (15.1)
Imipenem							
Sensitive	7 (6.7)	1 (14.3)	2 (28.6)	0 (0.0)	0 (0.0)	0 (0.0)	4 (57.1)
Resistant	98 (93.3)	47 (48.0)	16 (16.3)	6 (6.1)	11 (11.2)	1 (1.0)	17 (17.3)
Meropenem							
Sensitive	18 (15.7)	7 (38.9)	2 (11.1)	0 (0.0)	3 (16.7)	0 (0.0)	6 (33.3)
Resistant	87 (75.7)	41 (47.1)	16 (18.4)	6 (6.9)	8 (9.2)	1 (1.1)	15 (17.2)
Ceftazidime							
Sensitive	6 (20.7)	0 (0.0)	5 (83.3)	0 (0.0)	1 (16.7)	0 (0.0)	0 (0.0)
Resistant	23 (79.3)	0 (0.0)	13 (56.5)	0 (0.0)	10 (43.5)	0 (0.0)	0 (0.0)
Piperacillin-tazobactam							
Sensitive	15 (12.8)	6 (40.0)	0 (0.0)	0 (0.0)	5 (33.3)	0 (0.0)	4 (26.7)
Resistant	90 (77.1)	42 (46.7)	18 (20.0)	6 (6.7)	6 (6.7)	1 (1.1)	17 (18.9)
Minocycline							
Sensitive	17 (89.5)	0 (0.0)	17 (100.0)	0 (0.0)	0 (0.0)	0 (0.0)	0 (0.0)
Resistant	1 (5.3)	0 (0.0)	1 (100.0)	0 (0.0)	0 (0.0)	0 (0.0)	0 (0.0)
Not done	87 (5.3)	48 (55.2)	0 (0.0)	6 (6.9)	11 (12.6)	1 (1.1)	21 (24.1)
Aztreonam							
Sensitive	2 (18.2)	0 (0.0)	0 (0.0)	0 (0.0)	2 (100.0)	0 (0.0)	0 (0.0)
Resistant	9 (81.8)	0 (0.0)	0 (0.0)	0 (0.0)	9 (100.0)	0 (0.0)	0 (0.0)
Not done	94 (81.8)	48 (51.1)	18 (19.4)	6 (6.5)	0 (0.0)	1 (1.1)	21 (22.6)
Tobramycin							
Sensitive	2 (18.2)	0 (0.0)	0 (0.0)	0 (0.0)	2 (100.0)	0 (0.0)	0 (0.0)
Resistant	9 (81.8)	0 (0.0)	0 (0.0)	0 (0.0)	9 (100.0)	0 (0.0)	0 (0.0)
Not done	94 (81.8)	48 (51.1)	18 (19.4)	6 (6.5)	0 (0.0)	1 (1.1)	21 (22.6)
Polymyxin-B							
Sensitive	10 (8.3)	0 (0.0)	0 (0.0)	0 (0.0)	10 (100.0)	0 (0.0)	0 (0.0)
Resistant	1 (0.8)	0 (0.0)	0 (0.0)	0 (0.0)	1 (100.0)	0 (0.0)	0 (0.0)
Not done	94 (90.8)	48 (51.1)	18 (19.4)	6 (6.5)	0 (0.0)	1 (1.1)	21 (22.6)
Amoxyclav							
Resistant	78 (76.5)	47 (60.3)	3 (3.8)	6 (7.7)	0 (0.0)	1 (1.3)	21 (26.9)
Not done	27 (23.5)	1 (3.7)	15 (55.6)	0 (0.0)	11 (40.7)	0 (0.0)	0 (0.0)
Nitrofurantoin							
Sensitive	12 (12.9)	12 (100.0)	0 (0.0)	0 (0.0)	0 (0.0)	0 (0.0)	0 (0.0)
Resistant	7 (7.5)	6 (85.7)	1 (14.3)	0 (0.0)	0 (0.0)	0 (0.0)	0 (0.0)
Not done	86 (79.6)	30 (34.9)	17 (19.8)	6 (7.0)	11 (12.8)	1 (1.2)	21 (24.4)

The susceptibility of various organisms to antibiotics was assessed using Broth Microdilution (MIC, µg/mL) and Disc Diffusion (Zone Diameter, mm) methods. Among the tested organisms, *K. pneumoniae* (n=48) displayed a mean MIC of 0.82 ± 2.30 µg/mL and a mean zone diameter of 15.50 ± 3.78 mm, indicating a moderate level of susceptibility. *A. baumannii* (n=18) exhibited a higher mean MIC of 2.21 ± 5.02 µg/mL and a mean zone diameter of 14.39 ± 4.82 mm, suggesting reduced susceptibility to the tested antibiotics. *K. oxytoca* (n=6) displayed a relatively low mean MIC of 0.83 ± 0.61 µg/mL and a mean zone diameter of 15.33 ± 3.62 mm, indicating better susceptibility. *P. aeruginosa* (n=11) exhibited a mean MIC of 0.45 ± 0.22 µg/mL and a mean zone diameter of 16.09 ± 2.47 mm, showing good susceptibility. Notably, *C. freundii* (n=1) displayed a very low mean MIC of 0.25 ± 0.00 µg/mL and a zone diameter of 18.00 ± 0.00 mm, indicating high susceptibility to the tested antibiotics. *E. coli* (n=21) exhibited a mean MIC of 0.58 ± 0.50 µg/mL and a mean zone diameter of 14.90 ± 3.40 mm, indicating moderate susceptibility. These results provide valuable insights into the susceptibility profiles of these organisms, helping guide antibiotic therapy decisions in clinical settings (Table 3).

**Table 3:** Distribution of MIC and disc diffusion for gram negative bacteria in isolates of patients. MIC: minimum inhibitory concentration

Gram-Negative Bacteria	Broth Microdilution (MIC, µg/mL)	Disc Diffusion (Zone Diameter, mm)
	Mean ± SD	Mean ± SD
*Klebsiella pneumoniae* (n=48)	0.82 ± 2.30	15.50 ± 3.78
*Acinetobacter baumannii* (n=18)	2.21 ± 5.02	14.39 ± 4.82
*Klebsiella oxytoca* (n=6)	0.83 ± 0.61	15.33 ± 3.62
*Pseudomonas aeruginosa *(n=11)	0.45 ± 0.22	16.09 ± 2.47
*Citrobacter freundii* (n=1)	0.25 ± 0.00	18.00 ± 0.00
*Escherichia coli* (n=21)	0.58 ± 0.50	14.90 ± 3.40

The Broth Microdilution (MIC) method was employed to assess antibiotic susceptibility in various bacterial organisms. Among 48 *K. pneumoniae* isolates, 95.8% were sensitive, 2.1% showed intermediate susceptibility, and 2.1% were resistant. In 18 *A. baumannii* isolates, 88.9% were sensitive, and 10.1% were resistant, with no intermediate isolates. All six *K. oxytoca* isolates were sensitive. *P. aeruginosa* exhibited 100.0% sensitivity in 11 isolates. A single *C. freundii* isolate was 100.0% sensitive. Lastly, all 21 *E. coli* isolates showed sensitivity. These results emphasize the variable susceptibility patterns among different bacterial species, highlighting the need for tailored antibiotic treatments based on the infecting organism for effective management and to counter antibiotic resistance (Table 4).

**Table 4:** Distribution of MIC pattern for Gram-negative bacteria in isolates of patients. MIC: minimum inhibitory concentration

Gram-Negative Bacteria	Broth Microdilution (MIC, µg/mL) [Frequency (%)]		
	Sensitive	Intermediate	Resistant
*Klebsiella pneumoniae* (n=48)	46 (95.8)	1 (2.1)	1 (2.1)
*Acinetobacter baumannii* (n=18)	16 (88.9)	0 (0.0)	2 (10.1)
*Klebsiella oxytoca* (n=6)	6 (100.0)	0 (0.0)	0 (0.0)
*Pseudomonas aeruginosa* (n=11)	11 (100.0)	0 (0.0)	0 (0.0)
*Citrobacter freundii* (n=1)	1 (100.0)	0 (0.0)	0 (0.0)
*Escherichia coli* (n=21)	21 (100.0)	0 (0.0)	0 (0.0)

The antibiotic susceptibility profiles of various bacterial organisms were determined using the Disc Diffusion and VITEK 2 methods. For *K. pneumoniae*, 89.6% were sensitive and 10.4% were resistant by Disc Diffusion, while VITEK 2 indicated 95.8% sensitivity and 4.2% resistance. Among *A. baumannii* isolates, 83.3% were sensitive and 16.7% were resistant by Disc Diffusion, with VITEK 2 showing 88.9% sensitivity and 11.1% resistance. *K. oxytoca* isolates had 66.7% sensitivity and 33.3% resistance by Disc Diffusion, and 83.3% sensitivity and 16.7% resistance by VITEK 2. *P. aeruginosa* displayed 90.9% sensitivity and 9.1% resistance with Disc Diffusion and 100.0% sensitivity with VITEK 2. *C. freundii* was 100.0% sensitive by both methods. *E. coli* isolates showed 90.5% sensitivity and 9.5% resistance by Disc Diffusion and 100.0% sensitivity by VITEK 2. These findings emphasize variations in susceptibility patterns among bacterial species and the importance of using multiple methods for accurate antibiotic susceptibility testing (Table 5).

**Table 5:** Distribution of disc diffusion and VITEK 2 pattern for Gram-negative bacteria in isolates of patients.

Gram-Negative Bacteria	Disc Diffusion (Zone Diameter, mm)		VITEK 2	
	Frequency (%)			
	Sensitive	Resistant	Sensitive	Resistant
*Klebsiella pneumoniae* (n=48)	43 (89.6)	5 (10.4)	46 (95.8)	2 (4.2)
*Acinetobacter baumannii* (n=18)	15 (83.3)	3 (16.7)	16 (88.9)	2 (11.1)
*Klebsiella oxytoca* (n=6)	4 (66.7)	2 (33.3)	5 (83.3)	1 (16.7)
*Pseudomonas aeruginosa* (n=11)	10 (90.9)	1 (9.1)	11 (100.0)	0 (0.0)
*Citrobacter freundii* (n=1)	1 (100.0)	0 (0.0)	1 (100.0)	0 (0.0)
*Escherichia coli* (n=21)	19 (90.5)	2 (9.5)	21 (100.0)	0 (0.0)

The agreement between the results obtained from the VITEK 2 method and Disc Diffusion for antibiotic susceptibility testing was assessed for various bacterial organisms. Among the 48 isolates of *K. pneumoniae*, the agreement was 93.8%, with 89.6% being sensitive and 6.3% being resistant by Disc Diffusion, while VITEK 2 indicated 0% resistance. For *A. baumannii* isolates (n=18), the agreement was 94.4%, with 83.3% being sensitive and 5.6% being resistant by Disc Diffusion, compared to 11.1% resistance by VITEK 2. *K. oxytoca* isolates (n=6) showed an agreement of 83.4%, with 66.7% sensitivity and 16.7% resistance by both methods. *P. aeruginosa* isolates (n=11) had a high agreement of 90.9%, with 90.9% sensitivity and 9.1% resistance by Disc Diffusion, while VITEK 2 indicated 0% resistance. *C. freundii* displayed 100% agreement, with all isolates being sensitive by both methods. E. coli isolates (n=21) had an agreement of 90.5%, with 90.5% sensitivity and 9.5% resistance by Disc Diffusion, and no resistance by VITEK 2. These findings highlight good agreement between the two methods, emphasizing their reliability for antibiotic susceptibility testing across different bacterial species (Table 6).

**Table 6:** Level of agreement between disc diffusion and VITEK 2 pattern for Gram-negative bacteria in isolates of patients. DD: Disc Diffusion; S: sensitive; R: resistant

Gram-Negative bacteria			VITEK 2				Agreement (%)
			S		R		
			Frequency	%	Frequency	%	
*Klebsiella pneumoniae* (n=48)	DD	S	43	89.6	0	0.0	93.8
		R	3	6.3	2	4.2	
*Acinetobacter baumannii* (n=18)	DD	S	15	83.3	0	0.0	94.4
		R	1	5.6	2	11.1	
*Klebsiella oxytoca* (n=6)	DD	S	4	66.7	0	0.0	83.4
		R	1	16.7	1	16.7	
*Pseudomonas aeruginosa* (n=11)	DD	S	10	90.9	0	0.0	90.9
		R	1	9.1	0	0.0	
*Citrobacter freundii* (n=1)	DD	S	1	100.0	0	0.0	100.0
		R	0	0.0	0	0.0	
*Escherichia coli* (n=21)	DD	S	19	90.5	0	0.0	90.5
		R	2	9.5	0	0.0	

A comparative evaluation of antibiotic susceptibility testing methods, the gold standard (Broth Microdilution) versus Disc Diffusion, and the gold standard (Broth Microdilution) versus VITEK 2, demonstrates notable distinctions in their performance. In the comparison with Disc Diffusion, the Broth Microdilution method exhibits a sensitivity of 90.20%, implying its efficacy in correctly identifying susceptible isolates, but it also shows a lower negative predictive value (23.08%) and a moderate negative likelihood ratio (0.1), potentially leading to false negatives. Conversely, when compared to VITEK 2, the Broth Microdilution method showcases exceptional sensitivity (98.04%), along with perfect specificity, a very low negative likelihood ratio (0.02), a high positive predictive value (100.00%), and an elevated negative predictive value (60.00%). Moreover, the Broth Microdilution method’s accuracy remains strong (98.10%). These comparisons underscore the superior performance of the VITEK 2 system in identifying antibiotic susceptibility, especially regarding its sensitivity and negative predictive value, making it a robust choice for clinical settings where accuracy is paramount (Table 7).

**Table 7:** Sensitivity and specificity of disc diffusion and VITEK 2 with gold standard (broth microdilution) for Gram-negative bacteria in isolates of patients.

Gram-Negative Bacteria	Gold Standard (Broth Microdilution)	
	Sensitive	Resistant
VITEK 2		
Sensitive	100	0
Resistant	2	3
Sensitivity analysis	Value	95% CI
Sensitivity	98.04%	93.10% to 99.76%
Specificity	100.00%	29.24% to 100.00%
Negative likelihood ratio	0.02	0.00 to 0.08
Positive predictive value	100.00%	96.38% to 100.00%
Negative predictive value	60.00%	27.55% to 85.54%
Accuracy	98.10%	93.29% to 99.77%
Gram-negative bacteria	Gold standard (Broth Microdilution)	
	Sensitive	Resistant
Disc Diffusion		
Sensitive	92	0
Resistant	10	3
Sensitivity analysis	Value	95% CI
Sensitivity	90.20%	82.71% to 95.20%
Specificity	100.00%	29.24% to 100.00%
Negative likelihood ratio	0.1	0.05 to 0.18
Positive predictive value	100.00%	96.07% to 100.00%
Negative predictive value	23.08%	14.28% to 35.08%
Accuracy	90.48%	83.18% to 95.34%

## Discussion

The rising prevalence of antibiotic resistance among Gram-negative bacteria is a major concern for healthcare systems worldwide. This study aimed to investigate the antibiotic susceptibility patterns of various Gram-negative bacterial species in a clinical setting and evaluate the concordance between two commonly used susceptibility testing methods, the Kirby-Bauer Disc Diffusion method, and the VITEK 2 system. The findings of this study shed light on the extent of antibiotic resistance and the reliability of testing methods, providing valuable insights for clinical practice and antimicrobial stewardship.

In our study, the mean age of the participants was 47.30 years with a standard deviation of ± 21.46 years, indicating a wide age range in the sample. A similar mean age was reported in the studies by Taneja et al., Aggarwal et al., Arjun et al., and Goel et al., [13-16]. In our study, regarding the distribution of sexes, the study included 69 males (65.7%) and 36 females (34.3%), demonstrating a slight male predominance in the sample population. A similar pattern of sex distribution was observed in the studies by Taneja et al., Pragasam et al., Aggarwal et al., and Goel et al., [13,14,16,17]. In our study, the sample type analysis revealed that pus samples were the most common, constituting 27 cases (25.7%), followed by urine samples with 21 cases (20%). Other sample types, such as ETT and sputum, also had substantial representation. In other studies, by Zaki et al., Pawar et al., Bhaskar et al., and Jain, the pus samples and urine samples were the most common samples [18-21].

Our study identified several organisms responsible for infections, with *K. pneumoniae* being the most common (45.7%). *A. baumannii* accounted for 17.1% of cases, while other organisms like *E. coli* and *P. aeruginosa* were responsible for smaller proportions of infections. A similar spectrum of Gram-negative bacteria was noted in the studies by Behera et al., Bhaskar et al, Zilberberg et al., and Zaki et al., [4,18,20,22]. *P. aeruginosa* was the major Gram-negative bacteria in the studies by Pawar et al. and Jain, and *E. coli* was the major isolate in the study by Ramesh et al. [19,21,23].

Antibiotic resistance profiles

The results of this study revealed alarming rates of antibiotic resistance among Gram-negative bacterial isolates. *K. pneumoniae*, a frequent culprit in healthcare-associated infections, exhibited high levels of resistance to multiple antibiotics, with nearly 72.4% of isolates being resistant to ampicillin. These findings are consistent with the global trend of increasing resistance in *K. pneumoniae*, which poses a significant challenge in clinical management [17,18].

*A. baumannii*, another notorious multidrug-resistant pathogen, showed resistance to multiple antibiotics, with 10.1% of isolates being resistant to ampicillin. These results underscore the importance of robust infection control measures and the development of novel treatment strategies to combat *A. baumannii* infections [10,16].

Concordance of colistin susceptibility testing methods

One of the critical aspects of this study was the evaluation of the agreement between the Kirby-Bauer Disc Diffusion method and the VITEK 2 system. The results demonstrated substantial agreement between the two methods for several antibiotics, such as ciprofloxacin and amikacin, with percentage agreements exceeding 90%. This suggests that these antibiotics can be reliably tested using either method in clinical laboratories. The level of agreement varied between 80 and 90% in other studies by Singhal et al., Tan and Ng, Behera et al., Arroyo et al., Tan and Ng, Sinirtas et al., Behera et al., Maalej et al., and Dafopoulou et al., [4,7,12,24-29].

However, discrepancies were observed for certain antibiotics, emphasizing the need for cautious interpretation of susceptibility results. For instance, the VITEK 2 system classified a small proportion of isolates as resistant to antibiotics to which they were classified as sensitive by the Disc Diffusion method. These discrepancies may have clinical implications, particularly when selecting empirical antibiotic therapy, and it has been also shown in reported in the studies by Goldstein et al., Piewngam and Kiratisin, Arnold et al., Hindler and Humphries, Van Der Heijden et al., and Lo-Ten-Foe et al. [1,5,10,30-32]. Clinicians should be aware of the limitations of each testing method and consider patient-specific factors when interpreting results.

Clinical implications

The high prevalence of antibiotic resistance among Gram-negative bacteria highlights the urgency of implementing effective antimicrobial stewardship programs. Rational antibiotic use, tailored to local resistance patterns, is essential to preserve the efficacy of available antibiotics. Additionally, the findings of this study emphasize the need for continued research and development of new antimicrobial agents to combat multidrug-resistant Gram-negative infections.

Limitations

Several limitations should be acknowledged in our study. First, this research was conducted within the confines of a single healthcare facility, which may impose constraints on the generalizability of the findings. To enhance the external validity of our results, future research endeavors should consider multi-center studies that encompass diverse healthcare settings. Second, it is crucial to recognize potential sources of bias within our study. Measurement bias and selection bias may have influenced the results, and we acknowledge the need to address these biases explicitly. Additionally, the study focused primarily on antibiotic susceptibility testing methods and did not delve into the molecular aspects of resistance. Future research could explore the genetic mechanisms underlying resistance to provide a more comprehensive understanding of the topic. Last, variations in sample types were encountered, and while efforts were made to standardize protocols, the inherent differences in sample handling may have introduced variability in our results. Despite these limitations, our study contributes valuable insights into antibiotic resistance trends, and we recognize the importance of addressing these limitations in the interpretation of our findings.

## Conclusions

In conclusion, our study addresses the escalating challenge of antibiotic resistance among Gram-negative bacteria, offering insights into susceptibility patterns and testing methodologies. The mean age and sex distribution in our sample aligns with comparable studies, underscoring the demographic diversity. Noteworthy findings include the prevalence of pus and urine samples and the dominance of *K. pneumoniae* in causing infections, consistent with existing literature. The resistance profiles of *K. pneumoniae* and *A. baumannii* emphasize the pressing need for effective infection control strategies and novel treatment approaches.

The evaluation of colistin susceptibility testing methods revealed substantial agreement for various antibiotics, with certain discrepancies warranting careful interpretation. Clinicians must be mindful of method limitations, particularly when choosing empirical antibiotic therapy. Our study advocates for robust antimicrobial stewardship programs to combat the high prevalence of resistance. Despite limitations, such as single-center focus and potential biases, our findings contribute to understanding resistance trends. Future research should expand to diverse settings, explore molecular resistance mechanisms, and address variability in sample handling protocols. The imperative for rational antibiotic use and continual development of new agents to counter multidrug-resistant Gram-negative infections remains evident.
